# Accuracy and Readability of Artificial Intelligence Chatbot Responses to Vasectomy-Related Questions: Public Beware

**DOI:** 10.7759/cureus.67996

**Published:** 2024-08-28

**Authors:** Jonathan A Carlson, Robin Z Cheng, Alyssa Lange, Nadiminty Nagalakshmi, John Rabets, Tariq Shah, Puneet Sindhwani

**Affiliations:** 1 Urology, The University of Toledo College of Medicine and Life Sciences, Toledo, USA; 2 Urology, The University of Toledo Medical Center, Toledo, USA

**Keywords:** chat-gpt, chat gpt, readability measures, vasectomy knowledge, vasectomy, artificial intelligence ai

## Abstract

Purpose

Artificial intelligence (AI) has rapidly gained popularity with the growth of ChatGPT (OpenAI, San Francisco, USA) and other large-language model chatbots, and these programs have tremendous potential to impact medicine. One important area of consequence in medicine and public health is that patients may use these programs in search of answers to medical questions. Despite the increased utilization of AI chatbots by the public, there is little research to assess the reliability of ChatGPT and alternative programs when queried for medical information. This study seeks to elucidate the accuracy and readability of AI chatbots in answering patient questions regarding urology. As vasectomy is one of the most common urologic procedures, this study investigates AI-generated responses to frequently asked vasectomy-related questions. For this study, five popular and free-to-access AI platforms were utilized to undertake this investigation.

Methods

Fifteen vasectomy-related questions were individually queried to five AI chatbots from November-December 2023: ChatGPT (OpenAI, San Francisco, USA), Bard (Google Inc., Mountainview, USA) Bing (Microsoft, Redmond, USA) Perplexity (Perplexity AI Inc., San Francisco, USA), and Claude (Anthropic, San Francisco, USA). Responses from each platform were graded by two attending urologists, two urology research faculty, and one urological resident physician using a Likert (1-6) scale: (1-completely inaccurate, 6-completely accurate) based on comparison to existing American Urological Association guidelines. Flesch-Kincaid Grade levels (FKGL) and Flesch Reading Ease scores (FRES) (1-100) were calculated for each response. To assess differences in Likert, FRES, and FKGL, Kruskal-Wallis tests were performed using GraphPad Prism V10.1.0 (GraphPad, San Diego, USA) with Alpha set at 0.05.

Results

Analysis shows that ChatGPT provided the most accurate responses across the five AI chatbots with an average score of 5.04 on the Likert scale. Subsequently, Microsoft Bing (4.91), Anthropic Claude (4.65), Google Bard (4.43), and Perplexity (4.41) followed. All five chatbots were found to score, on average, higher than 4.41 corresponding to a score of at least "somewhat accurate." Google Bard received the highest Flesch Reading Ease score (49.67) and lowest Grade level (10.1) when compared to the other chatbots. Anthropic Claude scored 46.7 on the FRES and 10.55 on the FKGL. Microsoft Bing scored 45.57 on the FRES and 11.56 on the FKGL. Perplexity scored 36.4 on the FRES and 13.29 on the FKGL. ChatGPT had the lowest FRES of 30.4 and highest FKGL of 14.2.

Conclusion

This study investigates the use of AI in medicine, specifically urology, and it helps to determine whether large-language model chatbots can be reliable sources of freely available medical information. All five AI chatbots on average were able to achieve at least “somewhat accurate” on a 6-point Likert scale. In terms of readability, all five AI chatbots on average had Flesch Reading Ease scores of less than 50 and were higher than a 10th-grade level. In this small-scale study, there were several significant differences identified between the readability scores of each AI chatbot. However, there were no significant differences found among their accuracies. Thus, our study suggests that major AI chatbots may perform similarly in their ability to be correct but differ in their ease of being comprehended by the general public.

## Introduction

Natural language processing models have quickly gained popularity over the last few years with the growing utilization of artificial intelligence (AI) and its applicable uses to the public and beyond. These models can communicate with individuals by receiving inputs from text-based information and formulating a response derived from learned information [1]. As a result, these language models can interact in a conversational-type manner and function similarly to standard extant search engines. ChatGPT (Chat Generative Pre-Trained Transformer) is the most well-known model. ChatGPT was initially launched in November 2022 by OpenAI, and it has rapidly grown to become a well-known source of information, creativity, and inspiration [2]. This conversational chatbot is based on a large language model containing over 175 billion parameters, known as ChatGPT-3.5 [3].

ChatGPT-3.5 became considerably popular in 2023 when it was found to perform at the passing threshold on the United States Medical Licensing Exams (USMLE) [4]. This study provided evidence that ChatGPT was well-equipped to handle and respond to complex medical information; and, subsequent studies have shown that ChatGPT-4, a more up-to-date chatbot version, performed even better than previous models [5]. This has established ChatGPT as a competent model when interpreting and responding to medical questions [5]. These studies have helped to elucidate the vast potential and apparent deficiencies within artificial intelligence and its application in medicine. In addition to the performance of ChatGPT on board-style examination questions, studies have investigated its ability to respond to urological questions. Caglar et al. found that ChatGPT-3.5 could correctly respond to questions regarding pediatric urology 92.0% of the time [6]. Furthermore, another study by Caglar et al. provided evidence that ChatGPT-3.5 could respond to andrology-related questions satisfactorily 87.9% of the time [7]. Once again, this supports the evidence that ChatGPT can appropriately handle medical information and formulate correct responses.

While there is abundant evidence to highlight ChatGPT’s abilities and potential use in the medical field, there is a lack of research to evaluate its competitors. The recent technological explosion of AI has not gone unnoticed, and several other well-known companies have created alternative platforms that function similarly to ChatGPT.

Despite ChatGPT’s mainstream popularity, these alternative platforms warrant similar attention and investigation to compare their abilities, accuracy, and potential. As vasectomy is one of the most common urologic procedures, it serves as a valuable topic to evaluate these platforms and their ability to respond to questions that patients may pose. This study aims to explore the accuracy and readability of five commonly used AI chatbots: ChatGPT (OpenAI, San Francisco, USA), Bard (Google Inc., Mountainview, USA) Bing (Microsoft, Redmond, USA) Perplexity (Perplexity AI Inc., San Francisco, USA), and Claude (Anthropic, San Francisco, USA). ChatGPT-3.5 was selected because it is by far the most popular AI chatbot tool that exists today. Although the newest version, ChatGPT-4.0, has shown increasingly promising results in comparison to ChatGPT-3.5, it is locked behind subscription. Due to the subscription cost of ChatGPT-4.0, it is more likely that patients would query ChatGPT-3.5 to avoid these expenses. The remaining AI chatbots were selected for investigation based on both the popularity and accessibility of the programs.

## Materials and methods

Fifteen vasectomy-related questions were generated to represent some of the most commonly asked questions by patients prior to undergoing the procedure. These questions were produced by deliberation among a urology attending physician, a senior urology resident, and a medical student at the University of Toledo Medical Center. All questions were approved by the urology attending physician. Questions were worded using terminology and language expected of a layperson, as if the patients were utilizing the chatbots themselves. The list of questions is included in the Appendices (Table 2). An example AI-generated response from ChatGPT-3.5 is included in Appendices (Figure 2). From November to December 2023, evaluators queried 15 questions individually to five AI chatbots: ChatGPT-3.5, Google Bard rebranded as Gemini, Microsoft Bing Chat rebranded as Copilot, Perplexity AI, and Anthropic Claude. Each question was queried by starting a new chat-interaction on each of the platforms. The utilization of a new chat-interaction aids by preventing the AI platforms from adapting their responses based on multiple queries. Chatbot responses were recorded and extracted from their respective platforms and compiled into a list. These question-and-answer lists were then blinded and presented to the evaluators. Additionally, gold-standard responses were created for each of the 15 vasectomy-related questions using existing American Urological Association (AUA) guidelines and resources, and were approved by all urology attending physicians.

Accuracy was evaluated by comparison of AI-generated responses to the gold-standard responses by five blinded, independent evaluators: two attending urologists, two urology PhD research faculty, and one senior urology resident physician. By comparison to the gold-standard responses, evaluators critically analyzed the responses to assess how closely AI chatbot responses resemble the widely accepted information that is presented in the AUA guidelines. Using a Likert scale (1-6): (1-completely inaccurate, 2-mostly inaccurate, 3-somewhat inaccurate, 4-somewhat accurate, 5-mostly accurate, and 6-completely accurate), the evaluators graded the AI-generated responses. Every AI-generated response received an individual score given by each of the five evaluators. The average score and standard deviation for each chatbot were calculated using GraphPad Prism (GraphPad, San Diego, USA). The data was analyzed using Kruskal-Wallis as it is non-parametric. Kruskal-Wallis analysis was performed to compare the mean accuracy for each AI platform, using GraphPad Prism V10.1.0 with Alpha set at 0.05.

Readability was assessed using the Flesch-Kincaid Reading Ease Score (FRES) and Flesch-Kincaid Grade Level (FKGL) using an online readability calculator. Every AI-generated response was evaluated for both FRES and FKGL. The means for each AI chatbot was calculated. FRES provides a numerical value on a scale of (1-100), and a score between 60-70 is widely considered acceptable. The FKGL provides a numerical value that corresponds with the associated grade level, i.e. 8.2 would indicate a student with an 8th-grade reading level could understand the text. Kruskal-Wallis analysis was performed to compare the mean FRES and FKGL scores for each of the AI platforms, using GraphPad Prism V10.1.0 with Alpha set at 0.05. Kruskal-Wallis was chosen due to the non-parametric nature of the values.

## Results

The analysis included responses to fifteen questions asked in sequential order graded by five independent and blinded reviewers. The scores were averaged to derive mean response scores. 

Accuracy of information

The Likert scale was utilized to determine accuracy. The responses generated by ChatGPT-3.5 had the highest average score of 5.04, followed by Microsoft Bing with 4.91, Anthropic Claude 4.65, Google Bard 4.43, and last was Perplexity with a score of 4.41 (Table 1).

Readability

Flesch-Kincaid Reading Ease Score (FRES) and Flesch-Kincaid Grade Level (FKGL) were used to grade the readability of responses. Across all five AI search engines, FRES scores were all under the recommended score of 70. The average responses generated by Google Bard had the highest score of 49.67, followed by Anthropic Claude with 46.8, Microsoft Bing 45.57, Perplexity 36.4, and ChatGPT-3.5 with 30.4. FKGL assess grade level with a preferred grade reading level of 8. In these terms, Google Bard had the most preferred score with an average of 10.1, followed by Anthropic Claude with 10.55, Microsoft Bing 11.56, Perplexity 13.29, and ChatGPT-3.5 with 14.23 (Table 1).

**Table 1:** Summary of Endpoints reported as mean (± SD). FKGL: Flesch-Kincaid Grade Level; FRES: Flesch-Kincaid Reading Ease Score

TOOL	AI CHATBOT				
	Bing Chat	ChatGPT	Anthropic Claude	Google Bard	Perplexity AI
Likert	4.91 (± 0.81)	5.04 (± 0.67)	4.65 (± 0.97)	4.43 (± 1.09)	4.41 (± 1.12)
FRES	45.57 (±10.32)	30.4 (± 8.01)	46.8 (± 8.15)	49.67 (± 9.61)	36.4 (± 11.91)
FKGL	11.56 (± 1.91)	14.23 (± 1.50)	10.55 (± 1.52)	10.1 (± 1.56)	13.29 (± 1.55)

**Figure 1:**
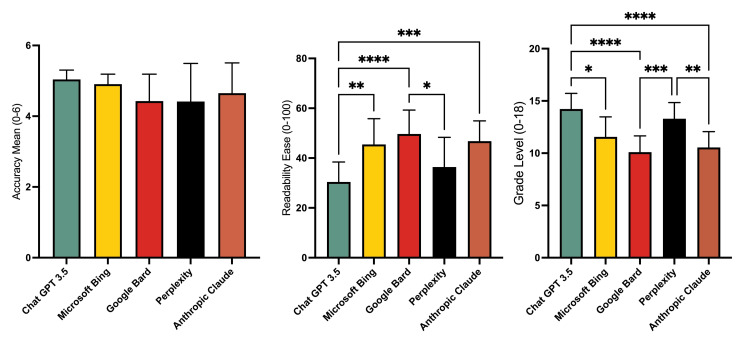
Kruskal-Wallis Analysis. A: Accuracy based on Likert scale, B: Flesch-Reading Ease Score, C: Flesch-Kincaid Grade Level. Bar graphs represent mean scores with standard deviation. Significant differences are noted with asterisks for various p-values. *, **, ***, and **** indicate p-values of ≤ 0.05, ≤ 0.01, ≤ 0.001, and ≤ 0.0001 respectively.

**Figure 2:**
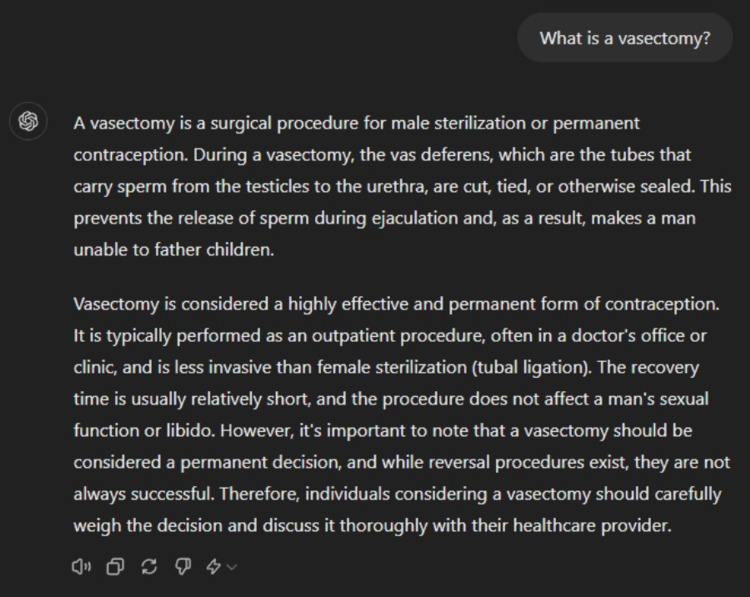
Example response from OpenAI's ChatGPT-3.5 to the question "What is a vasectomy?"

To assess differences in Likert, FRES, and FKGL, Kruskal-Wallis test was performed using GraphPad Prism V10.1.0 with Alpha set at 0.05. There were no significant differences in the Likert scale between the search engines (Figure 1). Regarding FRES, there were significant differences between ChatGPT-3.5 and Microsoft Bing, Google Bard, and Anthropic Claude (Figure 1). The final significant difference was between Google Bard and Perplexity. For FKGL, there were significant differences between ChatGPT, Microsoft Bing, Google Bard, and Anthropic Claude (Figure 1). Perplexity also had a significant difference with Google Bard and Anthropic Claude. It is important to note that the preferred grade level is < 8.

## Discussion

Artificial intelligence inherently comes with the premise to continuously advance and refine its abilities to better perform the tasks set before it. As a result, there are high expectations for all that these AI programs will be able to achieve. In the field of medicine, the use of AI has been an important area of study as it will inevitably be incorporated further into practice. Because of this, it is necessary for healthcare professionals to increase awareness and participation in this transition process. Furthermore, it is well known that the public may seek medical information using the internet. A 2005 study by Hesse, et al. found that 63.7% of the US population had utilized the world wide web to access information regarding the health of themselves or others [8]. Additionally, 48.6% of individuals in the study stated that they would pursue online medical information prior to talking with their physician [8]. In 2013, a survey conducted by the Pew Research Center confirmed that a substantial amount of the US population uses the internet for health information, and it was found that nearly two-thirds of respondents went online for this reason [9]. Since then, technology has only become more accessible, and it is reasonable to believe that this trend holds true today, if not with heightened incidence. With the exuberant growth of AI and ChatGPT, these platforms have become a potential location for the population to find medical information online, and it is necessary that these tools are assessed for their capabilities.

ChatGPT has been at the forefront of AI in medicine since it was released in November 2022. Since the time of its inception, there have been several studies to assess its applications in medicine. Initial studies investigated the capacity of this platform to handle medical information, using the USMLEs to gauge its abilities [4,5]. This was met with promising results that revealed that ChatGPT was able to reach the passing scores of these medical exams [4,5]. This data confirms that ChatGPT is certainly capable at processing and utilizing medical terminology and information. These studies helped to lay the groundwork for investigating and utilizing ChatGPT in medicine. Altogether, these studies have helped to identify the potential for use that ChatGPT and AI may ultimately possess.

Aside from AI’s ability to perform on medical exams, it is hypothesized that ChatGPT, and AI, have the potential to be used in the field of public health, and it is suggested that ChatGPT may hold value in functioning as a liaison of health information to the general public [10]. With regard to its use for public health, ChatGPT possesses several advantages that contribute to the potential of this tool. In an article by Dave, et al. it is evident that ChatGPT can be used as a rapid search engine with responses produced in a conversational and formally structured text [11]. Additionally, information obtained by the platform is accessed by analysis of available public literature [11]. Applicable uses of AI in medicine include ideas about healthy lifestyle choices, emphasis on vaccination importance, health screening suggestions, and environmental health questions [10]. Furthermore, ChatGPT can be used to explain the role of healthcare workers and provide information about community health programs [10]. However, it is of note that information provided by ChatGPT is susceptible to deficiencies such as limited accuracy, lack of context, and inherent biases [10,11]. The most concerning of these deficiencies is the lack of interaction with a health professional [10]. As a result, this highlights the importance of physicians increasing knowledge about AI in medicine and the importance of studies to investigate the validity of statements made by ChatGPT regarding health information.

With reference to AI in medicine, ChatGPT has consistently been the leading candidate for use, as evidenced by the substantial amount of literature highlighting its potential and capabilities. Despite this exciting prospect for AI, this has led to a lack of focus on alternative platforms that function with similar abilities. However, in one study by Lim, et al. ChatGPT 3.5, 4.0, and Google Bard were evaluated for accuracy, comprehensiveness, and self-correction [12]. These factors were scored with the use of a Likert scale, and it was found that ChatGPT 4.0 provided the most accurate responses, followed by ChatGPT 3.5 and Google Bard [12]. Additionally, there were no significant differences found between the comprehensiveness of each response, and ChatGPT 4.0 was crowned the most proficient chatbot of the three [12]. It is of note that ChatGPT 3.5 and Google Bard were both deemed to provide "Borderline" responses with "Poor" ratings in varying instances [12]. This study is one of the few that highlights the similarities and differences between alternative AI chatbots that exist today. Although this study includes Google Bard, there are several other AI platforms that exist and deservedly warrant our attention. Additionally, despite ChatGPT 4.0 being found to provide the most accurate responses, it must be ensured that responses concerning health information are delivered with a sensitivity to language that will be understood by those who are using it.

In a 2024 study, Ghanem, et al. investigated the quality of AI chatbots’ responses to questions regarding medical information on appendicitis. ChatGPT 3.5, ChatGPT 4.0, Google Bard, and Claude-2 were assessed for the quality of responses using a modified version of the DISCERN instrument [13]. It was found that Claude-2 was the only AI platform with significantly diminished quality when compared to the other AI chatbots [14]. Additionally, Ghanem, et al. also investigated the readability of each platform using FRES and FKGL [13]. In their study, they found that all AI platforms produced responses that were deemed difficult to read, but Google Bard was the most intelligible of the group [13]. It is of note that this more recent study takes yet another AI platform into consideration, and it continues to heighten awareness of the alternative platforms that exist. The study recommends watchful monitoring of these platforms as they continue to become more prevalent in the medical field.

This study helps to uncover any differences in both accuracy and readability between some of the most popular and well-known artificial intelligence platforms. The Likert scale aims to quantify evaluators' assessment of the correctness of each chatbot response in comparison to the standard of care. Here with regards to accuracy, each platform was able to hold its own against the others, as all platforms received scores on this scale that were graded at a minimum mean of (4) "Somewhat Accurate." ChatGPT-3.5 provided the highest accuracy score. However, data analysis demonstrated no significant differences in accuracy between the five AI chatbot engines. This indicates that despite receiving the highest score, other platforms were within reach of the standard set by ChatGPT.

It is essential to take note that ChatGPT-3.5 did have significantly worse FRES and FKGL scores when compared to alternative platforms. This may be due to medical jargon requiring a higher reading level for complete comprehension. This is demonstrated by ChatGPT having the highest needed grade level (FKGL) to understand at 14.23. This suggests that a reader would need to be able to read above a high-school level to fully comprehend the information thoroughly. Furthermore, ChatGPT-3.5 also has the lowest FRES score of 30.4, which could be related to using more accurate terms unfamiliar to the average American. Thus, it appears that increased accuracy in the utilization of ChatGPT may come at the cost of producing less reader-friendly information when compared to the other platforms. In attempting to crown one of these platforms as the "best," it is essential to take all these factors into consideration.

Additionally, it is noteworthy that all five AI platforms received FRES scores below the threshold of 50, indicating the text responses were graded as "difficult to read." This can potentially be explained by the nature of the questions being asked and the responses they generate. Medical terminology is inherently more complicated than the average American language, and all the AI platforms appeared to struggle to produce responses that would be considered acceptable in this format. Although the queried questions were created to be written from the patient's perspective, the chatbots have produced responses that may be difficult for them to understand. This concern can be alleviated by adequately prompting AI platforms to ensure the language is understandable and reader-friendly, as these platforms can adapt and conform to the writer’s direct request. However, it would be unlikely that a patient would pose such artificially-worded questions.

The readability of chatbot responses is an important criterion for their utilization. As the use of these AI chatbots continues to grow, it is only a useful program if patients can understand the wording and terminology with which it responds. For patients to trust the information they receive from AI chatbots, they must be able to comprehend the explanation. Zalzal, et al. conducted a ChatGPT-focused study in which questions posed in the patient perspective were graded by both experts in the field, and laypeople [14]. The level of education from laypeople participants varied from high school education to Masters degrees, and the authors found that laypeople felt confident in the answers provided by ChatGPT on average 79.8% of the time [14]. Of note, this superseded the expectations of the authors, and it suggests that the average American may be surprisingly trustworthy of these AI platforms. Additionally, they found that layperson-graders expressed that the AI responses were written with similar sophistication to their own doctors [14]. This highlights the importance of AI responses being both readable and understandable to the average patient. The application of laypeople in their study provides another layer of understanding of how the public may use and interact with AI for medical information. Of note, Zalzal, et al. did not include alternative AI platforms, and further research could help to identify if the public is more willing to trust answers generated from AI platforms they are familiar with [14]. Additionally, future analysis may help to reveal specific benefits to the use of alternative AI platforms, in comparison to ChatGPT.

Trusting information provided by AI platforms creates an ethical dilemma surrounding the usage of these programs. The platforms provide a unique and necessary increase to patient accessibility to medical information, but it naturally poses concern for the accuracy of information being produced. Studies have shown promising results in the performance of these chatbots, but they have not been without error. Thus, the usage of these chatbots deserves continued investigation to ensure the information provided is accurate. Patient accessibility to medical information is important, but it remains just as important to confirm the information is adequate. The inability to clarify the accuracy of AI chatbot responses opens the door to misinformation and negative consequences of their use. Thus, the use of these platforms should be cautioned until the validity of AI chatbot content is confirmed.

Limitations

Despite our results indicating that AI competitors are comparable to ChatGPT in terms of accuracy, this may result from limitations within the study. In drawing these conclusions, it is essential to note the limited sample size of questions. This is a small-scale study to compare these platforms, and although the results indicate no significant differences in accuracy, this may not hold with a larger data set. Further research is needed to assess the capabilities of these alternative platforms compared to ChatGPT, and studies should seek to evaluate how this continues to progress over time. Future studies on this topic should include a larger sample size of questions to determine the accuracy and readability of these AI chatbots. Furthermore, our study includes data from our queries posed to these AI chatbots in one instance. AI applications are known to provide variable responses to different iterations of the same question, and further investigation should seek to identify the consistency of AI chatbots and their responses to medical questions. Additional limitations to our study include the subjective nature of Likert scoring for accuracy. These scores were generated by comparison of AI chatbot responses to the gold-standard responses based on AUA guidelines, but it is a subjective measure. Thus, it is possible that evaluators have variability in their assessments of the accuracy using this scale. To date, there is no tool to assess the accuracy of these chatbot programs, and future research may seek to develop a tool for determining accuracy. As artificial intelligence continues to advance, multiple AI chatbots are being developed and constantly improved. The public may utilize any one of the AI chatbots, and thus, is imperative to continuously investigate the multitude of platforms to ensure that they are providing correct and comprehensible information to patients. 

## Conclusions

AI chatbots are becoming exceedingly prevalent in the medical space, and it is important to highlight their pitfalls and strengths as the general population increases their use. While most responses were relatively accurate, some were inadequate and difficult for the general public to understand. Relying on AI chatbots may provide convenience; however, difficulty in understanding the responses can be detrimental. Our findings suggest ChatGPT-3.5 is the most accurate, while Google Bard has the best readability score and desired reading grading level. It should be noted that these conclusions are based on our small-scale study, and that these results may not fully represent the abilities of these platforms in comparison. Patients should be aware of these shortcomings and continue to be encouraged to ask their physicians their medical questions or to clarify chatbot responses.

## Appendices

**Table 2:** 15 questions used to query AI chatbots.

Number	Question
1	What is a vasectomy?
2	How bad is the pain during a vasectomy?
3	What is the most common complication of a vasectomy?
4	Will vasectomy affect my sex drive?
5	How long is the recovery period after a vasectomy?
6	Am I sterile immediately after a vasectomy?
7	What is the failure rate of vasectomies?
8	Can a vasectomy be reversed?
9	What are the advantages of a vasectomy?
10	What are reasons to avoid having a vasectomy?
11	Will a vasectomy affect my ability to get an erection?
12	Will vasectomy affect ejaculation?
13	Will vasectomy affect urination?
14	When can I stop contraception after a vasectomy?
15	Does vasectomy cause cancer?
